# Clinical application of a GPU-accelerated monte carlo dose verification for cyberknife M6 with Iris collimator

**DOI:** 10.1186/s13014-024-02446-1

**Published:** 2024-07-02

**Authors:** Peng Zhou, Yankui Chang, Shijun Li, Jia Luo, Lin Lei, Yufen Shang, Xi Pei, Qiang Ren, Chuan Chen

**Affiliations:** 1 Department of Cancer Center, Daping Hospital, Army Medical University, Chongqing, China; 2 School of Nuclear Science and Technology, University of Science and Technology of China, Hefei, China; 3 Department of Radiation Oncology, Dezhou Second People’s Hospital, Dezhou, China; 4 Anhui Wisdom Technology Company Limited, Hefei, China

**Keywords:** GPU-accelerated Monte Carlo, CyberKnife, Patient-specific quality assurance

## Abstract

**Purpose:**

To apply an independent GPU-accelerated Monte Carlo (MC) dose verification for CyberKnife M6 with Iris collimator and evaluate the dose calculation accuracy of RayTracing (TPS-RT) algorithm and Monte Carlo (TPS-MC) algorithm in the Precision treatment planning system (TPS).

**Methods:**

GPU-accelerated MC algorithm (ArcherQA-CK) was integrated into a commercial dose verification system, ArcherQA, to implement the patient-specific quality assurance in the CyberKnife M6 system. 30 clinical cases (10 cases in head, and 10 cases in chest, and 10 cases in abdomen) were collected in this study. For each case, three different dose calculation methods (TPS-MC, TPS-RT and ArcherQA-CK) were implemented based on the same treatment plan and compared with each other. For evaluation, the 3D global gamma analysis and dose parameters of the target volume and organs at risk (OARs) were analyzed comparatively.

**Results:**

For gamma pass rates at the criterion of 2%/2 mm, the results were over 98.0% for TPS-MC vs.TPS-RT, TPS-MC vs. ArcherQA-CK and TPS-RT vs. ArcherQA-CK in head cases, 84.9% for TPS-MC vs.TPS-RT, 98.0% for TPS-MC vs. ArcherQA-CK and 83.3% for TPS-RT vs. ArcherQA-CK in chest cases, 98.2% for TPS-MC vs.TPS-RT, 99.4% for TPS-MC vs. ArcherQA-CK and 94.5% for TPS-RT vs. ArcherQA-CK in abdomen cases. For dose parameters of planning target volume (PTV) in chest cases, the deviations of TPS-RT vs. TPS-MC and ArcherQA-CK vs. TPS-MC had significant difference (*P* < 0.01), and the deviations of TPS-RT vs. TPS-MC and TPS-RT vs. ArcherQA-CK were similar (*P* > 0.05). ArcherQA-CK had less calculation time compared with TPS-MC (1.66 min vs. 65.11 min).

**Conclusions:**

Our proposed MC dose engine (ArcherQA-CK) has a high degree of consistency with the Precision TPS-MC algorithm, which can quickly identify the calculation errors of TPS-RT algorithm for some chest cases. ArcherQA-CK can provide accurate patient-specific quality assurance in clinical practice.

## Introduction

Supported by small field irradiation and precise positioning system, the implementation of CyberKnife (CK) could provide more precise and steeper dose distribution, which can deposit dose in the target area to a greater extent and reduce the dose of normal organs, so it is widely used for tumor treatment in clinical practice [1–5]. Bahig H et al. [6] compared the treatment effects of proton radiotherapy and CK in the head and neck region, the results indicated that CK could be a competitive alternative in large volume head and neck re-irradiation. Although CK has many unique and irreplaceable advantages, there are still some issues about dose calculation that need to be addressed. In CK system, small beam irradiation can easily cause electron imbalance in the beam. Currently, there are two dose calculation algorithms for Iris collimators available in the Precision™ Treatment Planning System, version 1.1.1.1 (Accuray, Wisconsin, USA), RayTracing (RT) algorithm and Monte Carlo (MC) algorithm. The RT algorithm uses effective path length to account for density variations [7], which ignores the scattering effects of anatomical structural heterogeneity and thus, the calculated dose in low-density regions is falsely high [8–10]. This may result in the actual dose transmitted to the patient being less than the prescription dose [11]. MC algorithm can fully consider the physical reactions during particle transport by simulating a large number of particles, including lateral electronic scatter and lateral electronic disequilibrium, so the MC-based dose calculation is regarded as the gold standard [12, 13]. However, the traditional MC algorithm requires a significant amount of computational time to get the ideal results, which may take several or even tens of hours. Therefore, CK system urgently needs a fast yet accurate dose calculation method.

Previous clinical researchers have already explored some works for comparison of dose calculation in CK system, including the comparison with measured dose and the development of third-party dose verification tools. Gondré M et al. [14] implemented the dose calculation of single beam in homogeneous and heterogeneous phantoms, multiple beams in homogenous and heterogeneous phantoms, and patient-specific dose verifications on clinical patients, the experimental results showed that the accuracy of MC model could fully meet clinical requirements. Heidorn SC et al. [15] built a heterogeneous phantom with lung-equivalent material and solid water for dose calculation, which demonstrated the accuracy of MC algorithm was much higher than the accuracy of correction-based Finite-Size Pencil Beam (FSPB) algorithm in low-density regions. Mackeprang PH et al. [16] proposed a vendor-independent MC dose calculation (IDC) framework as the ground truth to evaluate the accuracy of MC algorithm in precision TPS on 3 phantoms and 7 lung patients, indicating that Precision MC dose calculation was successfully benchmarked against IDC and measurements. Reynaert N et al. [17] performed a treatment plan QA platform based on BEAMnrc/DOSXYZnrc, which achieved high consistency with the MC algorithm of TPS, but the calculation times on 40 CPU cores are about 15 min for the CK using pre-calculated phase-space. Pan et al. [11] used the CIRS thorax phantom and film measurements to demonstrate that the RT algorithm performed well for homogeneous situation and failed for heterogeneous situation.

Numerous studies have demonstrated the accuracy of MC algorithm for CK system [11, 14–17], whether it is inherent in TPS or proposed by researchers. However, the above MC dose calculations are based on CPU cores and the computational time is still very long. The RT algorithm is mainly used in clinical practice. For this situation, it is necessary to carry out the patient specific quality assurance (QA) when the treatment plan is finished by RT algorithm. Therefore, the purpose of this study is to develop and validate a graphics processing unit (GPU) accelerated MC dose verification (ArcherQA-CK) for CyberKnife M6 system with Iris collimator. 30 clinical cases were collected for comparing the three different dose calculation methods (TPS-MC, TPS-RT and ArcherQA-CK), which were implemented based on the same treatment plan. Finally, the accuracy and efficiency of ArcherQA-CK are systematically analyzed.

## Materials and methods

### Datasets

In this study, we retrospectively collect 30 cases of cancer patients receiving CyberKnife treatments between September 2022 and April 2023 at our hospital (Army Medical Center of PLA in China), including 10 head cases, 10 chest cases and 10 abdomen cases. Three types of collimators are available in clinic, including fixed collimators with fixed diameters of 5–60 mm, Iris collimator with variable circular apertures of 5–60 mm and multi-leaf collimator (MLC). The cases treated in our center are mainly based on the Iris collimator. Table 1 shows the detailed information of 30 cases in this study, containing clinical diagnosis, treatment region, tracking methods, collimator size, prescription dose and number of fractions. For head cases, there are 7 cases with intracranial metastatic tumor and 3 cases with other head tumor, with the prescription dose from 16 Gy to 30 Gy. The volumes of planning target volume (PTV) range from 1.35cm^3^ to 42.68cm^3^. For chest cases, there are 9 cases with lung tumor and 1 case with metastatic tumor of the body (the target area is in the left upper lung), so the target areas of chest cases are all in the lungs, with prescription dose from 40 Gy to 60 Gy. The volumes of PTV range from 6.21cm^3^ to 91.81cm^3^. For abdomen cases, the target areas were mainly distributed in the kidney and pancreas regions, the prescription dose is ranging from 35 Gy to 60 Gy. The volumes of PTV range from 20.49cm^3^ to 191.89cm^3^. Due to the different sizes and distributions of target areas, each case was treated with Iris collimators with multiple variable circular apertures of 5–60 mm. All cases have passed clinical gamma testing and validation, and have completed the radiation therapy process. The clinical patient specific quality assurance was implemented on 1179 SRS MapCHECK (SunNuclear, Melbourne, USA), and gamma pass rates (2%/2 mm) all satisfied the requirements of clinical goal (over 95%).

**Table 1:** Detailed information of 30 cases in this study

Cases	clinical diagnosis	treatment region	tracking methods [18–20]	collimator size (mm)	prescription dose (Gy)	number of fractions
Head1	Intracranial metastatic tumor	head	6D-skull	10,15	17.5	5
Head2	Other head tumor	head	6D-skull	10,15,25	21	3
Head3	Intracranial metastatic tumor	head	6D-skull	15,25,40	20	5
Head4	Intracranial metastatic tumor	head	6D-skull	12.5,20	18	2
Head5	Other head tumor	head	6D-skull	10,15	16	2
Head6	Intracranial metastatic tumor	head	6D-skull	10	18	2
Head7	Intracranial metastatic tumor	Left cerebellum + rear corner of right ventricle + right occipital	6D-skull	12.5,20	18	3
Head8	Intracranial metastatic tumor	head	6D-skull	10,20,35	20	5
Head9	Other head tumor	head	6D-skull	12.5,20	18	2
Head10	Intracranial metastatic tumor	head	6D-skull	12.5	30	10
Lung1	Lung tumor	left upper lung	Xsight-Lung	10,20,30	54	4
Lung2	Lung tumor	left lower lobe	Xsight-Spine	10,12.5,20	50	5
Lung3	Lung tumor	right lower lobe	Xsight-Spine	25,35,50	55	5
Lung4	Lung tumor	right upper lobe anterior segment	Xsight-Spine	15,25,35	60	5
Lung5	Lung tumor	right Middle Lobe	Xsight-Lung	15,30,50	60	5
Lung6	Lung tumor	right upper pulmonary hilum	Xsight-Spine	12.5,30,40	56	7
Lung7	Lung tumor	right lung	Xsight-Spine	12.5,20,30	40	5
Lung8	Lung tumor	right lung	Xsight-Spine	20,30,40	56	7
Lung9	Metastatic tumor of the body	Left upper lung	Xsight-Spine	15,25	50	5
Lung10	Lung tumor	right upper pulmonary hilum	Xsight-Spine	12.5,20,25	55	6
Abdomen1	Metastatic tumor of the body	right adrenal gland	Synchrony	40,50,60	50	5
Abdomen2	pancreatic tumor	pancreatic	Xsight-Spine	20,35,50	35	5
Abdomen3	pancreatic tumor	abdominal cavity	Xsight-Spine	12.5,20	35	5
Abdomen4	renal cell carcinoma	left renal pelvis	Xsight-Spine	12.5,25	40	5
Abdomen5	Metastatic tumor of the body	left mediastinum	Xsight-Spine	15,25	50	5
Abdomen6	Metastatic tumor of the body	right mediastinum	Xsight-Spine	15,25	50	5
Abdomen7	Metastatic tumor of the body	left adrenal gland	Xsight-Spine	15,30	40	5
Abdomen8	pancreatic tumor	pancreatic	Xsight-Spine	15,35	56	8
Abdomen9	Metastatic tumor of the body	left adrenal gland	Xsight-Spine	25,35,50	45	5
Abdomen10	Metastatic tumor of the body	Liver + adrenal gland	Xsight-Spine	12.5,15,25	40	5

### ArcherQA-CK

Monte Carlo dose calculation is considered the gold standard, but with more computational time. We have made plenty of works [21–26] on Monte Carlo dose calculations and integrated the codes into an online third-party QA system for secondary dose verification of radiation treatment plans, ArcherQA, which can realize multiple functions such as delivery logfile analysis, alignment accuracy checks and three-dimensional (3D) gamma analysis. Based on ArcherQA, we recently developed a GPU-accelerated Monte Carlo dose verification for CyberKnife M6 system, named ArcherQA-CK, to provide efficient and accurate patient specific quality assurance (PSQA) in clinical practice.

The particle transport process of ArcherQA-CK follows the definition of ArcherQA, where photoelectric effect, Compton scattering, and Rayleigh scattering can take place. It is worth emphasizing that the calculation process is accelerated by GPU parallel computing processing, which can quickly obtain dose calculation results. The source model of ArcherQA-CK MC algorithm is constructed based on Francescon’s work [27], where the simulation of the treatment head of CK used geometry and material composition provided by the manufacturer with the BEAMnrc code [28]. The energy, position, direction, and other information of the particles after the secondary collimator are recorded to form the phase space. During the process of adjusting the source model, the measured dose was used as the reference, including percent dose depth (PDD) curves in water tank with a size of 60 mm collimator and off-center ratio (OCR) curves in water tank at 10 cm depth for each size of collimator (projection diameter: 5 mm, 7.5 mm, 10 mm, 12.5 mm, 15 mm, 20 mm, 25 mm, 30 mm, 35 mm. 40 mm, 50 mm, 60 mm). When measuring PDD, the source skin distance (SSD) is 800 mm. When measuring OCR, the source to detector distance is always 800 mm. The acquisition of these data is performed with a PTW 60,017 stereotactic diode (PTW, Germany) in a water tank (MEPHYSTO mcc 3.3). The uncertainty of ArcherQA-CK (defined by the average relative standard deviation of each voxel) in water tank is 1% and the calculation resolution is 1 mm. The absolute dose in ArcherQA-CK is performed that 1 MU = 1 cGy at 800 mm SAD (source-to-axis distance) and 15 mm depth with the 60 mm collimator.

International Atomic Energy Agency (IAEA) provides the phase space file with collimator of 60 mm [29], if directly used in ArcherQA-CK, the PDD curve of ArcherQA-CK matches well with the measured PDD curve. However, the OCR curve of ArcherQA-CK differs significantly from the measured OCR curve. To correct this situation, we divide the projection plane into 60 regions with a radial distance of 0 to 60 mm and a 1 mm interval. At 10 cm depth, the measured values of different regions are divided by the simulated values to obtain the radial correction coefficient. For each primary particle, its weight multiplies by the radial correction coefficient of its projection along a straight line to the region where the plane is located. Repeat this operation and continuously update the radial correction coefficient to make the measured value of OCR at this depth as close as possible to the simulated value, thereby obtaining the phase space after passing through the 60 mm collimator.

For other sizes of collimator (except for 60 mm collimator), the radius is R and four correction factors are used (δR_1_, δR_2_, W_1_, W_2_) to modify the phase space mentioned above. Firstly, the weight of particles with a projection radius (R_p_) greater than R is set to 0, there is a significant difference in the simulated OCR in the penumbral dose drop area. The simulated value in the area within radius R is greater than the measured value, while the simulated value outside radius R is smaller than the measured value. Therefore, for particles with a velocity ratio greater than 0.99 in the depth direction, the weight is changed to W_1_ if they satisfy ${R}_{p}\in [R,R+\delta {R}_{1}]$, the weight is changed to W_2_ if they satisfy $[R-\delta {R}_{2},R]$. The four correction factors are obtained by continuously simulating and comparing the OCR curves at 10 cm depth.

### Experiment

**Fig. 1:**
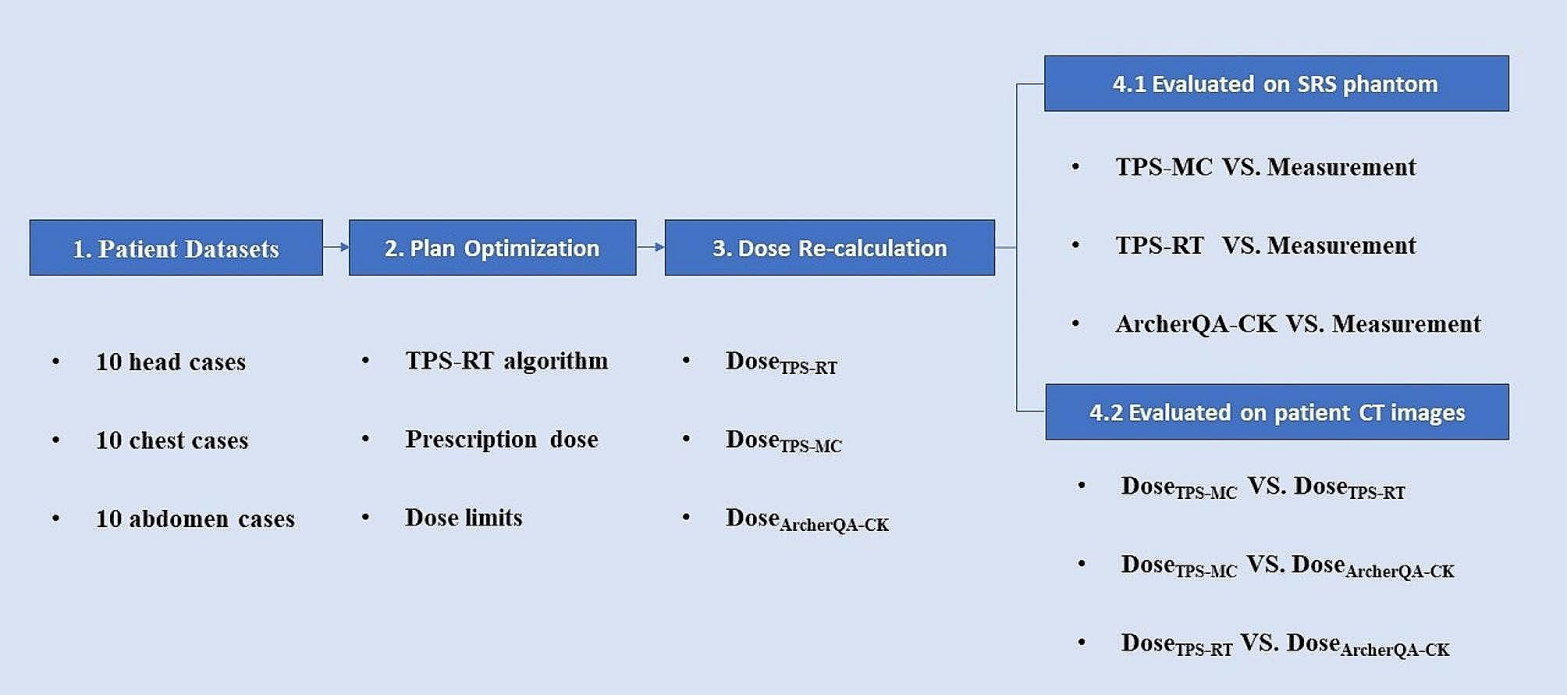
Flowchart of the experiment. 10 head cases, 10 chest cases, and 10 abdominal cases were collected firstly. Then, the plan was generated according to the prescription dose and other dose limits, optimized by RT algorithm in TPS. Furthermore, the dose recalculations were implemented by TPS-RT, TPS-MC and ArcherQA-CK algorithms to get three dose distributions. Finally, the differences of three dose distributions were compared

In the CyberKnife M6 radiosurgery system arranged in our hospital, there are two dose calculation algorithms, RayTracing algorithm (TPS-RT) and MC algorithm (TPS-MC). The flowchart of the experiment is shown in Fig. 1, the procedure can be divided into four steps. First, 10 head cases, 10 chest cases, and 10 abdominal cases were collected. Second, the plan was optimized by TPS-RT algorithm with the prescription dose and dose limits, because the TPS-RT algorithm can be completed with less time. Third, the dose re-calculations were implemented on patient CT images by TPS-RT, TPS-MC and ArcherQA-CK to get three different dose distributions (Dose_TPS−RT_, Dose_TPS−MC_, Dose_ArcherQA−CK_), where the uncertainty of MC dose calculation was set to 0.5% and the dose calculation resolution was about 1 mm ×1 mm ×1 mm. In this study, the Dose_TPS−RT_ and Dose_TPS−MC_ were calculated on the Precision TPS, Dose_ArcherQA−CK_ was obtained with ArcherQA-CK according to the beam information stored in the RT plan files. Finally, the three algorithms were evaluated with the measurements on SRS phantom. In addition, the differences of three dose distributions were compared with each other on 30 cases. In addition, the computational time of two MC algorithms (TPS-MC and ArcherQA-CK) was recorded for comparing the efficiency of different MC algorithms.

### Evaluation metrics

The global 3D gamma analysis (using an absolute dose comparison and 10% low-dose threshold) was completed with PTW Verisoft software, version 5.1 (PTW, Frieburg, Germany), using the following criteria: 2%/1 mm, 3%/1 mm, 2%/2 mm, and 2%/3 mm. In addition, the voxel-wise dose difference within the body was evaluated according to the formula, ΔD = D_g_ – D_c_, where D_g_ means the dose values of one dose distribution and D_c_ means dose values of another dose distribution.

In order to further reflect the impact of different dose distributions on the structures, the dosimetric parameters of target area and organs at risk were statistically evaluated and analyzed, including D_mean,_ D_2_ and D_95_ of PTV (here D_i_ means the dose received by i% of PTV volume), as well as D_mean_ of organs at risk. Paired sample T tests were used to evaluate the statistical significance of all the dose-volume parameters.

## Results

**Fig. 2:**
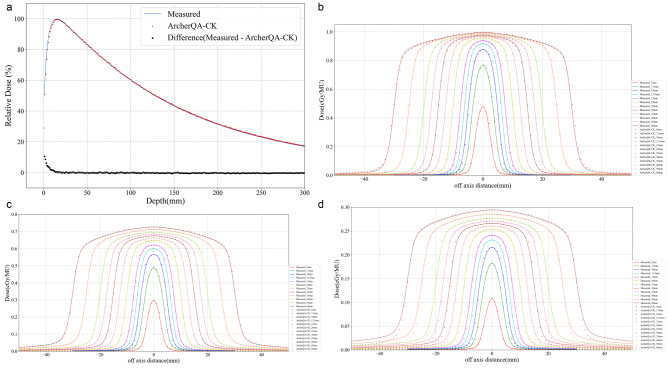
Dose comparison of ArcherQA-CK and measurements on water tank. (**a**) Percentage depth dose curve with 60 mm collimator. (**b**) Off-center ratio (OCR) curves with for 12 collimators at 1.5 cm depth. (**c**) Off-center ratio (OCR) curves with for 12 collimators at 10 cm depth. (**d**) Off-center ratio (OCR) curves with for 12 collimators at 30 cm depth

Figure 2(a) shows the diagram of percentage depth dose curves in water tank with a size of 60 mm collimator, Fig. 2(b-d) show off-center ratio (OCR) curves in water tank at depths of 1.5 cm, 10 cm, and 30 cm for each size of collimator. The curves of ArcherQA-CK calculated dose and measured dose almost coincide, which indicates that the simulated results by ArcherQA-CK is correct compared with the measured dose. Table 2 shows the global 3D gamma pass rates of three dose calculation algorithms compared with the measurements on SRS phantom for 30 cases, the average gamma pass rates for all dose calculation algorithms are greater than 97% at 2%/2 mm. According to the clinical requirements, the dose calculation results can be executed if the measurement on SRS phantom is used for plan check.

**Table 2:** Gamma pass rates (Ave ± Std) of three dose calculation algorithms compared with the measurements on SRS phantom for 30 cases (threshold = 10%, global 3D)

	2%/2 mm (%)	2%/3 mm (%)
TPS-MC	99.5 ± 0.9	99.9 ± 0.1
TPS-RT	99.2 ± 1.0	99.9 ± 0.4
ArcherQA-CK	97.2 ± 3.8	98.9 ± 2.2

Tables 3, 4 and 5 show the gamma pass rates of TPS-MC vs. TPS-RT, TPS-MC vs. ArcherQA-CK and TPS-RT vs. ArcherQA-CK on head, chest and abdomen cases, respectively. Overall, the ArcherQA-CK algorithm and TPS-MC algorithm have high consistency and the average gamma pass rates for head, chest and abdomen cases are greater than 98% at 2%/2 mm. Compared with the two MC algorithms, the built-in TPS-RT algorithm has achieved fairly good performance in head cases, followed by the abdomen cases, and performs the worst in chest cases. At the criteria of 2%/2 mm, the gamma pass rates are 98.8% ± 2.4% (TPS-MC vs. TPS-RT), 99.6% ± 0.4% (TPS-RT vs. ArcherQA-CK) for head cases, 98.2% ± 6.1% (TPS-RT vs. TPS-MC) and 94.5%±7.4%(TPS-RT vs. ArcherQA-CK)for abdomen cases, 84.9% ± 12.1% (TPS-MC vs. TPS-RT) and 83.3% ± 11.7% (TPS-RT vs. ArcherQA-CK) for chest cases, respectively. The gamma pass rate of TPS-MC vs. ArcherQA-CK is significantly higher than that of TPS-MC vs. TPS-RT in chest cases(*P* < 0.01).

**Table 3:** Gamma pass rates (Ave ± Std) of three dose calculation algorithms for 10 head cases (threshold = 10%, global 3D)

	2%/1 mm (%)	3%/1 mm (%)	2%/2 mm (%)	2%/3 mm (%)
TPS-MC vs. TPS-RT	98.3 ± 2.9	98.9 ± 2.2	98.8 ± 2.4	98.7 ± 2.1
TPS-MC vs. ArcherQA-CK	98.5 ± 2.2	99.4 ± 1.2	99.4 ± 1.4	99.6 ± 1.0
TPS-RT vs. ArcherQA-CK	97.4 ± 1.5	98.7 ± 0.8	99.6 ± 0.4	99.8 ± 0.2
P^*^ value	0.85	0.54	0.27	0.23

^*^P was calculated by comparing the gamma pass rates of TPS-MC vs. TPS-RT and TPS-MC vs. ArcherQA-CK according to paired sample t tests

**Table 4:** Gamma pass rates (Ave ± Std) of three dose calculation algorithms for 10 chest cases (threshold = 10%, global 3D)

	2%/1 mm (%)	3%/1 mm (%)	2%/2 mm (%)	2%/3 mm (%)
TPS-MC vs. TPS-RT	78.4 ± 15.2	84.2 ± 12.4	84.9 ± 12.1	87.3 ± 9.3
TPS-MC vs. ArcherQA-CK	96.3 ± 5.6	97.6 ± 5.0	98.0 ± 4.4	98.6 ± 3.6
TPS-RT vs. ArcherQA-CK	73.9 ± 14.4	82.3 ± 12.2	83.3 ± 11.7	88.5 ± 8.6
P^*^ value	0.002	0.005	0.001	0.001

^*^P was calculated by comparing the gamma pass rates of TPS-MC vs. TPS-RT and TPS-MC vs. ArcherQA-CK according to paired sample t tests

**Table 5:** Gamma pass rates (Ave ± Std) of three dose calculation algorithms for 10 abdomen cases (threshold = 10%, global 3D)

	2%/1 mm (%)	3%/1 mm (%)	2%/2 mm (%)	2%/3 mm (%)
TPS-MC vs. TPS-RT	93.6 ± 9.7	98.5 ± 2.0	98.2 ± 6.1	99.3 ± 4.9
TPS-MC vs. ArcherQA-CK	98.2 ± 2.7	99.1 ± 1.6	99.4 ± 1.1	99.7 ± 0.5
TPS-RT vs. ArcherQA-CK	86.5 ± 13.2	94.9 ± 4.9	94.5 ± 7.4	95.9 ± 4.7
P^*^ value	0.19	0.45	0.06	0.05

^*^P was calculated by comparing the gamma pass rates of TPS-MC vs. TPS-RT and TPS-MC vs. ArcherQA-CK according to paired sample t tests

Table 6 summarizes the comparison of dosimetric parameters for different anatomical structures, where the three dose distributions were compared by TPS-RT vs. TPS-MC, TPS-RT vs. ArcherQA-CK and ArcherQA-CK vs. TPS-MC. Overall, the deviations of ArcherQA-CK vs. TPS-MC are smaller than that of TPS-RT vs. TPS-MC, especially for the dosimetric parameters of target area in chest cases with significant difference (*P* < 0.01). For PTV in head cases, the deviations of ArcherQA-CK vs. TPS-MC and the deviations of TPS-RT vs. TPS-MC are both less than 1 Gy, and there is no significant difference. For chest cases, the dose calculated by TPS-RT is relatively high, and the average deviations of TPS-RT vs. TPS-MC in PTV dosimetric parameters are 9.5 Gy (PTV_D_95_), 7.02 Gy (PTV_D_2_), and 8.43 Gy (PTV_D_mean_), respectively, and the values of ArcherQA-CK vs. TPS-MC are 0.91 Gy (PTV_D_95_), 1.38 Gy (PTV_D_2_), and 1.05 Gy (PTV_D_mean_), respectively. For organs at risk in chest cases, the deviations of ArcherQA-CK vs. TPS-MC and TPS-RT vs. TPS-MC are both less than 1 Gy. For abdomen cases, the deviations of ArcherQA-CK vs. TPS-MC is comparable to that of TPS-RT vs. TPS-MC, with an average deviation of about 1 Gy for PTV and less than 0.5 Gy for organs at risk. There is no significant difference in the deviation between the RT algorithm and the two MC algorithms (P2 > 0.05).

**Table 6:** Dosimetric parameter deviations of different dose calculation methods (Gy)

Anatomical site	Evaluation indicators	TPS-RT vs. TPS-MC		TPS-RT vs. ArcherQA-CK			ArcherQA-CK vs. TPS-MC		*P*1 value*	*P*2 value*
		range of differences (MIN, MAX)	Average difference*	range of differences (MIN, MAX)	Average difference*	range of differences (MIN, MAX)		Average difference*		
Head	PTV_D_95_	(-0.75, 0.25)	0.3	(-1.15, 0.71)	0.49	(-0.48, 0.70)		0.27	0.77	0.72
	PTV_D_2_	(-1.09, 0.17)	0.4	(-3.37, 0.55)	1.09	(-0.38, 2.28)		0.73	0.17	0.13
	PTV_D_mean_	(-0.83, 0.19)	0.33	(-1.61, 0.59)	0.62	(-0.41, 0.89)		0.33	0.99	0.62
Chest	PTV_D_95_	(3.58, 22.96)	9.5	(3.94, 21.01)	9.37	(-2.21, 1.95)		0.91	**< 0.01**	**0.95**
	PTV_D_2_	(2.29, 16.47)	7.02	(3.52, 17.22)	7.92	(-3.01, 1.26)		1.38	**< 0.01**	**0.67**
	PTV_D_mean_	(3.31, 21.0)	8.43	(3.72, 20.09)	8.87	(-2.80, 1.32)		1.05	**< 0.01**	**0.84**
	Aorta_D_mean_	(-0.08, 0.39)	0.12	(0.02, 0.35)	0.19	(-0.23, 0.06)		0.07	0.19	0.38
	Heart_D_mean_	(-0.28, 0.47)	0.2	(-0.05, 0.54)	0.21	(-0.23, 0.04)		0.05	0.01	0.72
	Left Lung_D_mean_	(-0.02, 0.73)	0.3	(0.11, 0.72)	0.31	(-0.15, 0.05)		0.05	< 0.01	0.82
	Right Lung_D_mean_	(-0.01, 0.81)	0.33	(0.05, 0.91)	0.38	(-0.24, 0.1)		0.07	0.02	0.71
Abdomen	PTV_D_95_	(-0.18, 1.86)	0.81	(-0.01, 3.09)	1.87	(-2.27, -0.17)		1.09	0.52	0.07
	PTV_D_2_	(0.03, 2.06)	0.93	(0.41, 1.9)	1.76	(-1.23, 0.44)		0.92	0.99	0.08
	PTV_D_mean_	(-0.16, 1.92)	0.86	(-0.01, 2.66)	1.82	(-1.90, -0.17)		0.99	0.69	0.12
	Duodenum_D_mean_	(-0.21, 0.97)	0.36	(-0.13, 1.75)	0.49	(-1.11, 0.15)		0.16	0.2	0.60
	Stomach_D_mean_	(-0.15, 1.06)	0.34	(-0.004, 1.15)	0.36	(-0.28, 0.01)		0.09	0.04	0.63
	Left Kidney_D_mean_	(-0.01, 1.08)	0.32	(0.03, 1.03)	0.44	(-0.12, 0.15)		0.05	0.04	0.97
	Right Kidney_D_mean_	(-0.06, 1.06)	0.33	(0.01, 1.0)	0.4	(-0.08, 0.07)		0.04	0.03	0.95

*: Average difference means the average of the absolute values of the difference

*:*P*1 was calculated by comparing TPS-RT vs. TPS-MC and ArcherQA-CK vs. TPS-MC

*P*2 was calculated by comparing TPS-RT vs. TPS- MC and TPS-RT vs. ArcherQA-CK

**Fig. 3:**
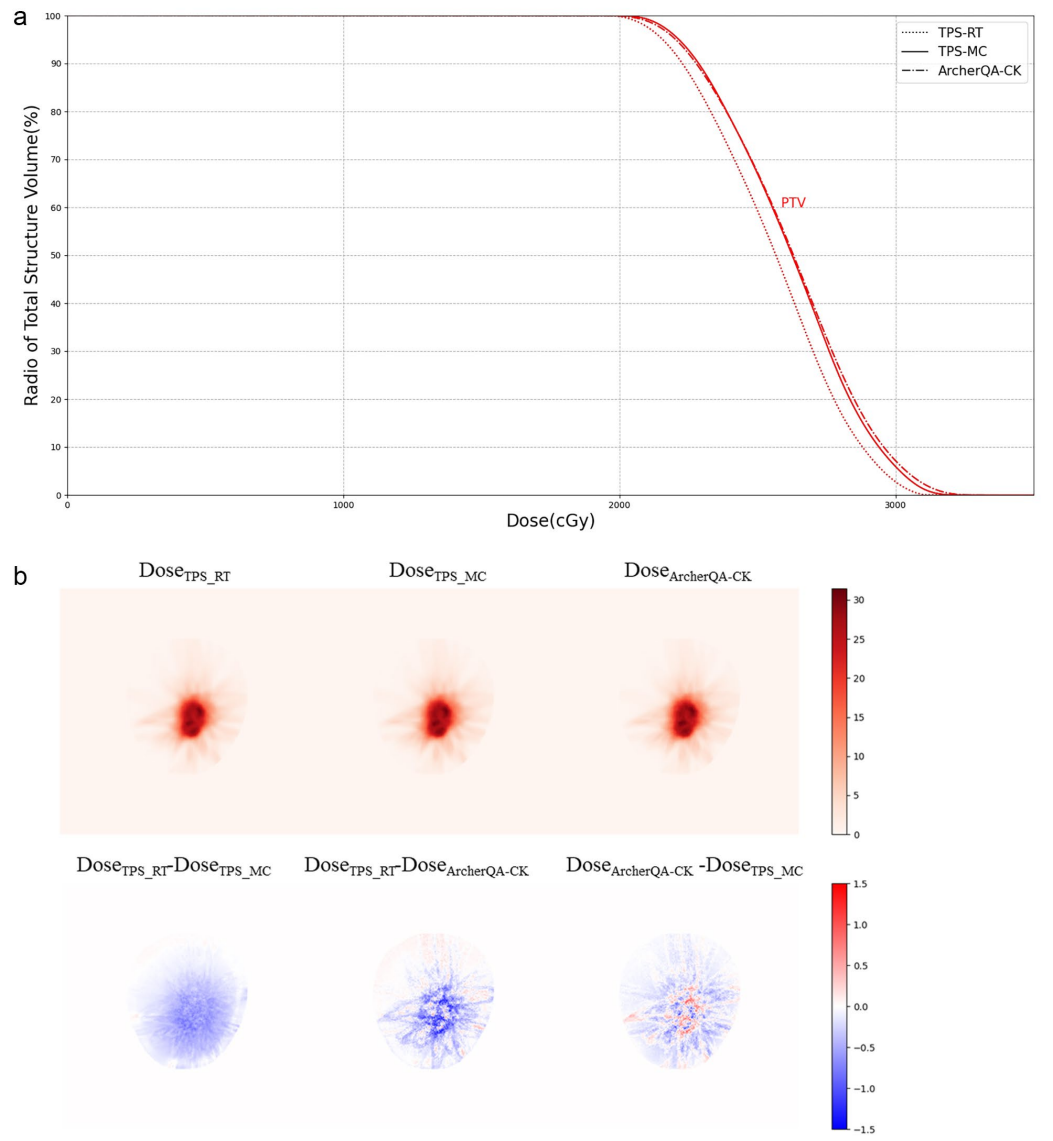
The dose-volume histogram comparison of planned target volume and dose distributions for a randomly selected head case. (**a**) Dose-volume histogram. The three curves are drawn based on three different dose distributions, namely TPS-MC (solid), TPS-RT (dotted) and ArcherQA-CK (dashdot). (**b**) Dose distributions and differences

**Fig. 4:**
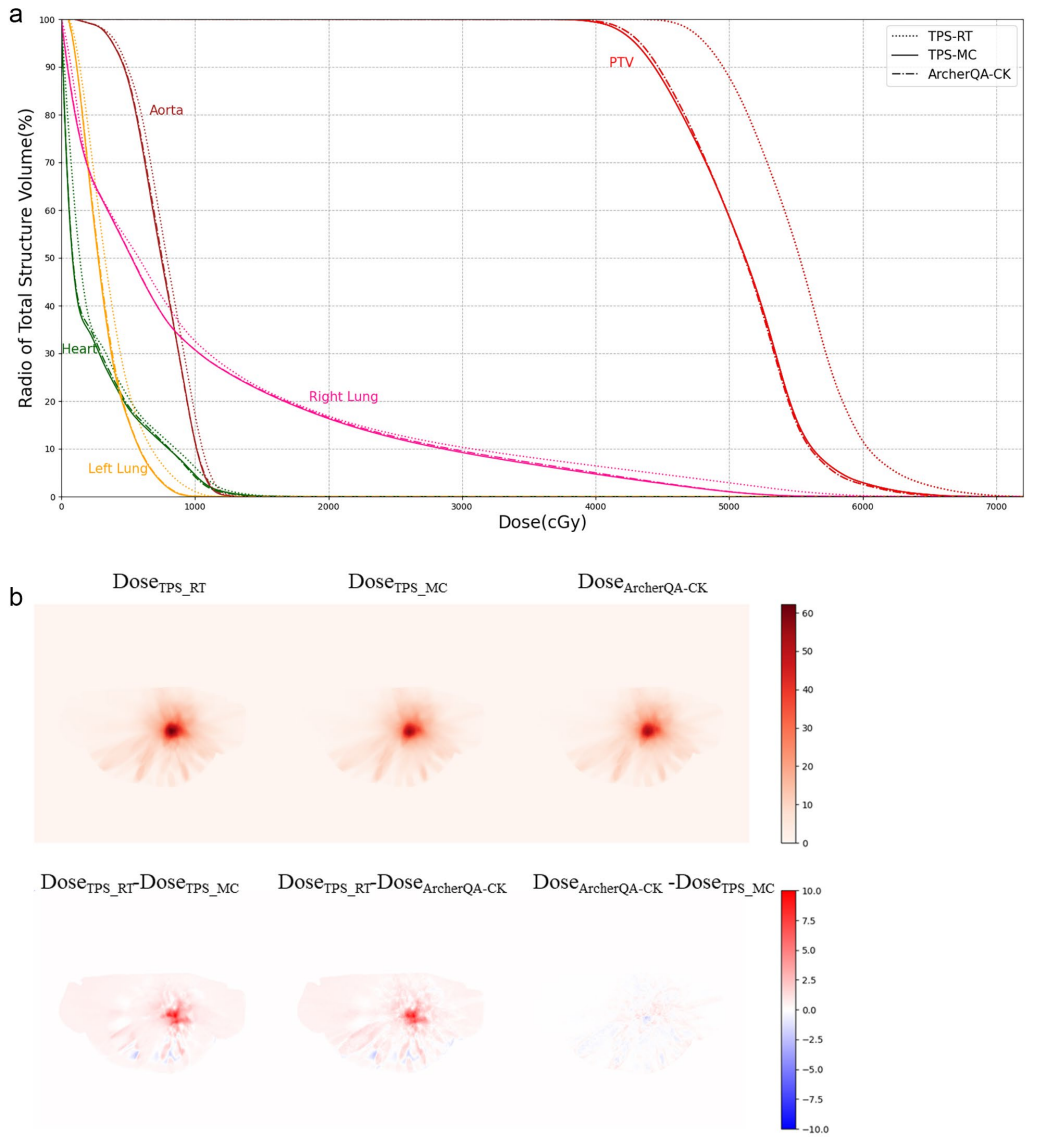
The dose-volume histogram comparison and dose distributions for a randomly selected chest case. (**a**)Dose-volume histogram of organs at risk and planned target volume. The three curves are drawn based on three different dose distributions, namely TPS-MC (solid), TPS-RT (dotted) and ArcherQA-CK (dashdot). (**b**)Dose distributions and differences

**Fig. 5:**
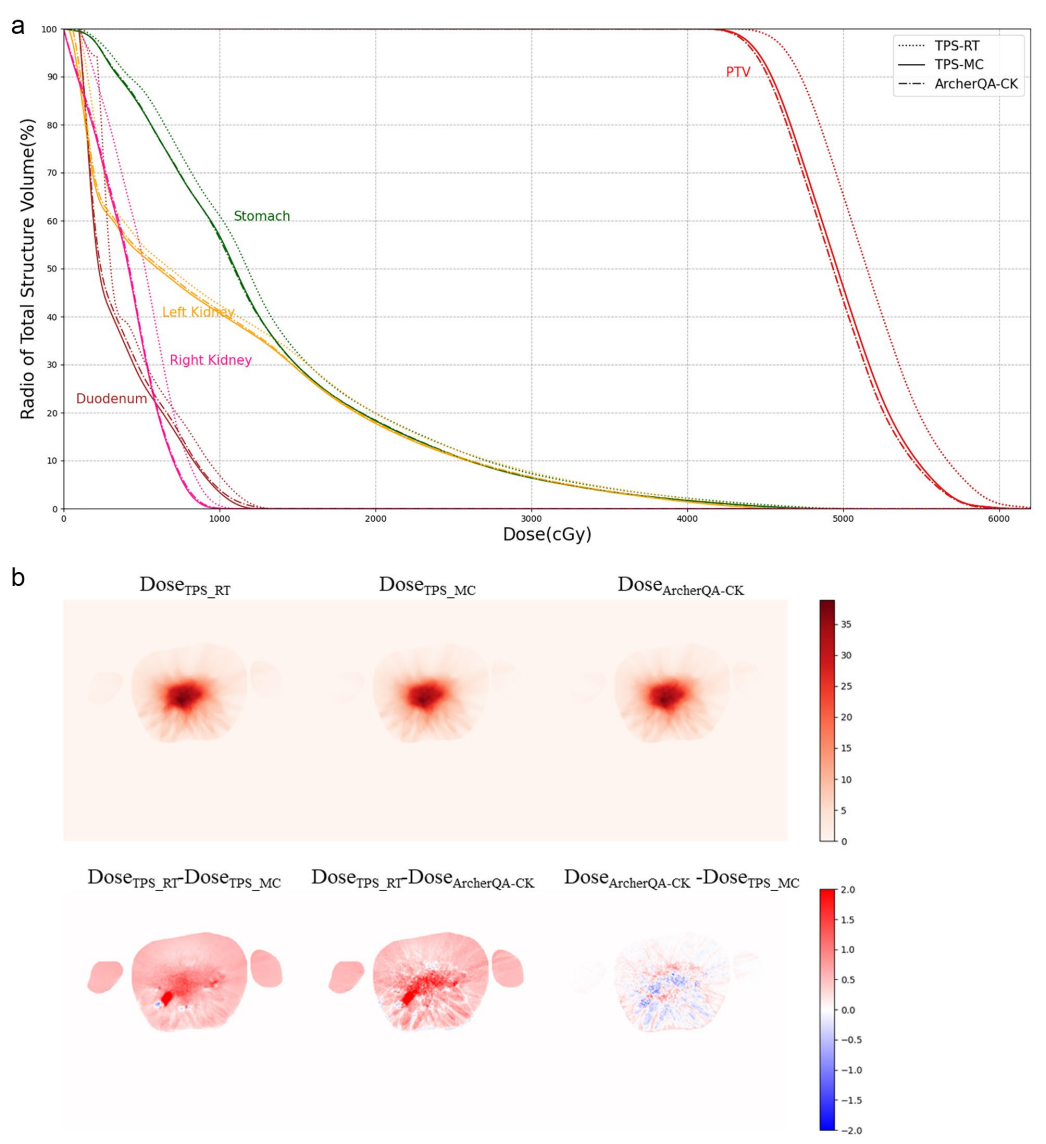
The dose-volume histogram comparison and dose distributions for a randomly selected abdomen case. (**a**) Dose-volume histogram of organs at risk and planned target volume. The three curves are drawn based on three different dose distributions, namely TPS-MC (solid), TPS-RT (dotted) and ArcherQA-CK (dashdot). (**b**)Dose distributions and differences

Figures 3, 4 and 5 show the dose-volume histogram (DVH) comparisons and dose distributions of a head case, a chest case and an abdomen case. The head case has the intracranial metastatic tumor, the prescription dose is 20 Gy. Due to the distance between the organs at risk and the target area, only PTV’s DVH is plotted. Figure 3(a) shows that the DVH curves of the two MC methods are very close, and DVH curve of the TPS-RT is relatively large, the result is consistent with the result in Table 3. The randomly selected chest case in Fig. 4 is central adenocarcinoma of the upper right lung combined with small cell carcinoma with multiple bone metastases, whose target area is located in right lung, the prescription dose is 56 Gy, the structures for comparison include PTV, aorta, heart, left lung and right lung. In Fig. 4(a), it can be seen that the DVH curve of TPS-RT is relatively high, and the difference for PTV is particularly significant, which is similar with the results of Table 6. The DVH curves of two MC methods are close in the chest case. The randomly selected abdomen case is central adenocarcinoma in the lower lobe of the right lung with metastasis to the right hilar and mediastinal lymph nodes, as well as metastasis to the right pleura, intracranial, and left adrenal glands. The target area is in left kidney and the prescription dose is 45 Gy, the structures for comparison include PTV, duodenum, stomach, left kidney and right kidney. Figure 5(a) shows that the difference of two MC methods is smaller than the difference between MC method and TPS-RT method.

The dose distributions calculated by the two Monte Carlo methods are very close. In addition, we recorded the calculation time of the two MC methods with an uncertainty of 0.5%. For the 30 cases in this study, the time spent by TPS-MC was 3906.6s ± 2370.8s, and the time spent by ArcherQA-CK was 99.8s ± 77.1s. The time of TPS-MC and ArcherQA-CK for head, chest and abdomen cases was 2722.1s ± 1410.7s vs. 42.3s ± 16.7s, 4423.3s ± 3036.2s vs. 109.5s ± 66.2s and 3633.4s ± 1393.7s VS. 138.7s ± 87.2s, respectively. From the comparison of calculation time, the efficiency of ArcherQA-CK was increased by about 39 times compared to TPS-MC.

## Discussion

There are two dose calculation methods in the Precision CyberKnife M6 system, Raytracing algorithm and Monte Carlo algorithm, which have their own advantages and disadvantages in clinical practice. In this study, we proposed a GPU-accelerated Monte Carlo dose calculation tool, ArcherQA-CK, for Cyberknife M6 system. We compared the three dose calculation methods (TPS-MC, TPS-RT and ArcherQA-CK) according to the same beam information for head, chest and abdomen cases, and the results showed that the dose distribution calculated by ArcherQA-CK was consistent with that calculated by TPS-MC. The accuracy of the TPS-RT algorithm in the head and abdomen cases is acceptable, but there is a significant difference in the dose distributions calculated by the TPS-RT algorithm and MC algorithm in the chest cases, especially in the high dose area near the target area. Our proposed ArcherQA-CK is based on GPU parallel computing, which can complete the dose calculation process within 2 min, providing the possibility for MC-based quality assurance in clinical practice.

Dose verification is necessary in clinical practice, and the 30 cases in this study have all passed the verification using the measured dose on phantom, which can be also seen in Table 2. The gamma pass rates for TPS-MC, TPS-RT and ArcherQA-CK algorithms are acceptable according to the clinical requirements. However, the gamma pass rates of TPS-RT and MC algorithms are small in chest cases, which is less than 95% at the criterion of 2%/3 mm. That is to say, the dose transmission deviation may occur even the dose verification has been completed and passed with the phantom. The main reason for this situation is the dose calculation inaccuracy of RayTracing algorithm on heterogeneous materials. From the data in Table 6, the Raytracing algorithm in the Precision TPS overestimates the dose distributions for chest and abdomen cases, with the most significant performance in the dosimetric parameters of PTV. The DVH curves in Fig. 3(a) and Fig. 4(a) also illustrate the overestimation. Sharman et al. [30] mentioned in their research that traditional analysis algorithm would overestimate the dose delivered to low-density heterogeneous volumes, and the level of overestimate depends on the field size and energy. Pan et al. [11] evaluated the accuracy of RT algorithm using the measured dose in phantom as comparison, their results showed that the RT algorithm would cause the delivered dose less than the prescription dose, so the MC algorithm was recommended in low-density heterogeneous regions. As shown in Table 6, the max difference of TPS-RT and TPS-MC are 22.96 Gy, 16.47 Gy and 21 Gy for D_95_, D_2_ and D_mean_ of PTV in chest cases, respectively. In abdomen cases, the max difference of TPS-RT and TPS-MC are 1.86 Gy, 2.06 Gy and 1.92 Gy for D_95_, D_2_ and D_mean_ of PTV, which are smaller than the difference in chest cases. There is almost no difference for TPS-MC and TPS-RT in head cases. Taking the chest case in Fig. 4 as an example, the prescription dose is 56 Gy, and the D_95_ of PTV calculated using the RT algorithm is 56 Gy, which can satisfy the requirement of D95 ≥ 56 Gy. However, the D_95_ of PTV calculated by Precision MC is only 49.9 Gy due to the overestimation of dose distribution by the RT algorithm, which means that the actual delivered dose is far less than the planned dose. The overestimation can lead to insufficient target irradiation and even treatment failure. Meanwhile, the D_95_ of PTV calculated by ArcherQA is 50.2 Gy, which is close the result of Precision MC, this situation can be avoided if ArcherQA-CK can be used for QA after using RT algorithm calculation. Therefore, the MC algorithm is recommended in chest cases, or at least used to complete the planning quality assurance.

The Monte Carlo method is considered as the gold standard for dose calculation, but the Monte Carlo algorithm in the Precision treatment system is very time-consuming, with an average calculation time of 65 min on 30 cases. Although many studies have proven the accuracy of the Precision Monte Carlo method, the use of Monte Carlo in clinical practice with such a long calculation time is very difficult. We proposed a GPU-accelerated Monte Carlo calculation method that can complete dose calculation within 2 min, providing a QA reference mode for clinical practice. In the Cyberknife M6 system, the dose gradient is large, so the dose delivered to the surrounding organs is relatively low. According to our treatment experience, it is generally to first optimize a plan with RT algorithm and then normalize it to ensure that the dosimetric parameters in the target area of the plan meet clinical goals. If the dose calculation is inaccurate, such as the dose overestimation phenomenon of RT algorithm for chest case in this study, it will result in an incorrect normalization factor, which will lead to insufficient target irradiation. According to the results in this paper, the RT algorithm in the TPS system can be used for head patients for consideration of dose calculation time and accuracy. However, it is best to use Monte Carlo method for chest and abdomen cases to complete the plan design. At least, Monte Carlo dose calculation should be used to recalculate the dose of the plan, ensuring the clinical goals have been achieved.

There are still some limitations in this study that need further improvement in subsequent work. Firstly, we have implemented the MC model with the Iris collimator due to the limitations of our hospital’s machines. In the future, we will continue to work with other hospitals to verify the dose for MLC Cyberknife treatment. Secondly, patient data from three locations were selected to verify the accuracy of the two dose calculation methods in the precision TPS system. Furthermore, the applicability of the two dose calculation methods in different tumor types can be determined in the future.

## Conclusion

In this study, we proposed a GPU-accelerated Monte Carlo dose calculation engine suitable for CyberKnife M6 system (ArcherQA-CK), and comprehensively compared the accuracy of ArcherQA-CK with the TPS-RT algorithm and TPS-MC algorithm in Precision TPS on head, chest, and abdomen clinical patients. The results indicate that the consistency between ArcherQA-CK and TPS-MC is very high, superior to the TPS-RT algorithm in the TPS system, especially in chest cases. While achieving high accuracy, ArcherQA-CK can complete dose calculation within 2 min. ArcherQA-CK can be served as a third-party independent dose calculation verification tool to provide patient specific quality assurance for clinical practice.

## Data Availability

The raw data supporting the conclusions of this article will be made available by the authors, without undue reservation.

## Abbreviations

CK: CyberKnife
MC: Monte Carlo
GPU: Graphics Processing Unit
RT: RayTracing
TPS: Treatment Planning System
FSPB: Finite-Size Pencil Beam
IDC: Independent Dose Calculation
QA: Quality Assurance
PSQA: Patient Specific Quality Assurance
